# The Identical External Reference Point Standardized to the Zero-Reference Level for Measuring Both Central and Jugular Venous Pressures: An Observational Study

**DOI:** 10.1155/2022/7329863

**Published:** 2022-12-19

**Authors:** Niraj Karmacharya, Madhur Dev Bhattarai, Amita Pradhan

**Affiliations:** ^1^Department of Medicine, Civil Service Hospital, Kathmandu, Nepal; ^2^Department of Medicine, Chitwan Medical College, Bharatpur, Nepal; ^3^Department of Community Dentistry, People's Dental College and Hospital, Kathmandu, Nepal

## Abstract

**Background:**

Studies report discrepancies between CVP and JVP measurements. The mid-thoracic plane (MTP) at the anterior fourth intercostal space level indicates the zero-reference level (ZRL) for venous pressure measurement, and the midaxillary line (MAL) at fourth intercostal space is a point near the ZRL in the supine position. JVP is usually measured from the sternal angle (SA) with further addition of 5 cm (JVP-SA + 5) and CVP in the supine position from MAL (CVP-MAL). However, no report has compared CVP measured from MTP (CVP-MTP) with CVP-MAL and with JVP from MTP (JVP-MTP) and JVP-SA + 5.

**Methods:**

We measured JVP-MTP and JVP-SA + 5 in appropriate reclining positions and subsequently CVP-MTP and CVP-MAL in the supine position blindly in 150 patients. We compared the pressures by Pearson correlation and Bland–Altman plots.

**Results:**

CVP-MTP and CVP-MAL demonstrated similar means (*p* = 0.129), strong positive linear relationship (*r* = 0.908), and good agreement (near-zero mean difference) with each other. JVP-MTP was about 1 cm higher than JVP-SA + 5 (*p* < 0.001). JVP-MTP displayed higher correlation coefficients and better agreements with both CVPs than JVP-SA+5. Correlation coefficients and mean differences of both CVPs with JVP-MTP were almost equal, about 0.83 and 1 cm, and with JVP-SA + 5 also almost equal, about 0.72 and 2 cm, respectively.

**Conclusions:**

JVP tallies better with CVP examined in the supine position when both are measured from MTP as the identical external reference point (ERP), and MAL can be used as MTP to measure CVP in the supine position. Our findings indicate the way to explore the matching of CVP and JVP to the full extent possible by standardizing their measurements from other identical ERPs to that from the zero-reference level MTP. Their further study in similar higher reclining positions from identical ERPs, such as MTP, MAL, and SA with the addition of higher numbers instead of 5 cm, is warranted standardizing other measurements to that from MTP.

## 1. Introduction

Central venous pressure (CVP) refers to the mean vena caval or right atrial pressure [1] and can be estimated by the physical examination of jugular venous pressure (JVP) [2]. The phlebostatic axis or zero reference level for venous pressure measurements is considered to be around the right atrium (posterior or center part) or the tricuspid valve [1, 3–10]. Venous pressures of healthy adults measured from the zero-reference level vary less than 1 to 2 cm with various body positions [1]. In any posture, the zero-reference level is indicated by the horizontal plane through the midpoint of the anteroposterior diameter of the thorax, midthoracic plane (MTP), at the anterior fourth intercostal space-level [3, 8, 11, 12]. The midaxillary line (MAL) at the fourth intercostal space is a point near the zero-reference level in the supine position [1]. CVP is usually measured in the supine position from MAL as the external reference point of the zero-reference level [1, 9]. Similarly, JVP is clinically measured from the sternal angle (SA), and 5 cm, as the vertical distance between the SA and zero-reference level, is added to the measurement to make the JVP equal to CVP [1]. However, studies in general report discrepancies between the CVP and JVP measurements [1, 2, 13–20], which may be related to the factors associated with either or both of them. Due to this, it may not be possible to utilize the JVP, the noninvasive method of CVP estimation, fully in critical patients. The determinants of the discrepancy between CVP and JVP measurements may provide insights into their complementary and appropriate use in critical care.

Deol et al. reported in 2011 that JVPs measured by clinical and ultrasound collapse pressure examinations were about 5 cm lower than CVP, with both CVP and JVP measurements calibrated as the total vertical distance from MAL after initially measuring them from SA [13]. In a previous study reported in 2020 from our department, the JVP measured from MTP (JVP-MTP) demonstrated a higher correlation coefficient with the CVP measured in the supine position from MAL (CVP-MAL) than the JVP from SA with further addition of 5 cm (JVP-SA + 5) [14]. CVP measured through an indwelling central venous catheter is the gold standard of venous pressure [2]. There is, however, hardly any report comparing the CVP measured from the zero-reference level MTP with the CVP from MAL and with the JVP from MTP and SA. We hypothesized that CVP measured in the supine position from MTP (CVP-MTP) would have a strong correlation and good agreement with CVP-MAL and would have a better correlation and agreement with JVP-MTP than with JVP-SA + 5. We correlated CVP-MTP and CVP-MAL, both measured in the supine position, with each other and with JVP-MTP and JVP-SA + 5 clinically measured in appropriate reclining positions and also studied the agreements between them.

## 2. Materials and Methods

We performed an observational study in intensive care units (ICUs) of the National Academy of Medical Sciences (NAMS) Hospitals, Kathmandu, Nepal, between January 2013 and February 2014. Conscious, spontaneously breathing adult subjects who already had a central venous catheter as part of their clinical management were potentially eligible for the study. We included in our study the patients with visible right internal jugular venous pulsations for JVP measurement and with the tip of the central venous catheter confirmed by a chest X-ray to be at the level of the carina [21, 22]. We excluded patients with neck or body radiotherapy history, chronic obstructive pulmonary diseases, chest wall deformity, large cervical lymph node, goiter causing venous distension, or superior ven`a cava obstruction. All consecutive eligible subjects were approached, and informed consent was obtained. The institutional review board of the NAMS approved the study (reference number 518/2071/72).

JVP was measured from two external reference points: the midthoracic plane (MTP) and sternal angle (SA). The MTP from the anterior fourth intercostal space to the back was marked using a ruler in the right axilla. The bed angle was adjusted for JVP measurement to yield the best views of right internal jugular venous pulsations. The observer used tangential lighting to recognize jugular venous pulsations and could strip the veins as required. The vertical distance from the marked point of MTP in the right axilla to the apex of the right internal jugular venous pulsations at end-expiration was measured as the JVP from MTP (JVP-MTP). Similarly, the vertical distance from the SA to jugular venous pulsation was measured, and 5 cm was added to obtain the value of JVP-SA + 5.

The first author measured the JVPs by physical examination with the help of the JVP Meter, which was also used in the previous study [14]. Maintaining the perpendicularity of horizontal and vertical rulers may not be accurate in the traditional two-ruler method to measure JVP from SA [23], and errors may also occur while measuring venous pressure directly at a time from the external reference points such as MAL in the axilla [13]. JVP Meter has been designed to directly measure the shorter or longer vertical distance from the external reference points such as MTP, MAL, and SA without using the two-ruler method. It has a movable top horizontal plane arm to indicate the upper level of jugular venous pulsations and a base indicator arm to indicate the external reference points such as MTP and SA. Both parts are attached at the right angle to the central frame that is kept upright and movable on a supporting stand, and the vertical distance between the top horizontal plane and base indicator arms can be directly read as the JVP from the central frame calibrated in centimeter [14].

The first author marked the MTP in the right axilla and measured the JVP-MTP and JVP-SA + 5. Then, the ICU physicians independently measured the CVP in the supine position from the marked point of MTP as CVP-MTP and from MAL as CVP-MAL. They were blinded to each other's measurements of JVP and CVP. CVP was measured with a water column manometer. The upper level of the water column in the manometer was used to measure the CVP, and the average value between the swings observed with respiration was recorded [24, 25].

We calculated the mean, standard deviation (SD), and confidence interval (CI) of CVP-MTP, CVP-MAL, JVP-MTP, and JVP-SA + 5. We compared the CVP-MTP and CVP-MAL with each other and with the JVP-MTP and JVP-SA + 5 graphically using Pearson correlation and Bland–Altman plot and calculated the correlation coefficient and mean difference (mean bias) with limits of agreement. A significance level of 5% was used, and 95% CIs were reported.

## 3. Results

**Figure 1:**
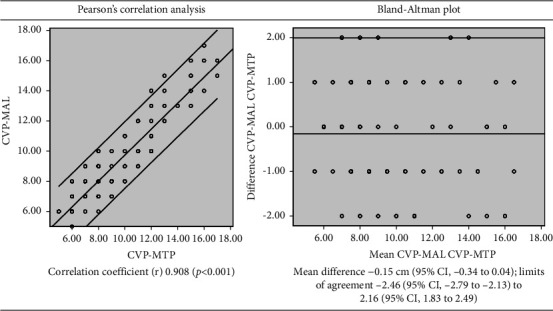
Pearson's correlation and Bland–Altman plots between CVP-MAL and CVP-MTP of 150 subjects. CVP-MAL: central venous pressure (CVP) measured from the midaxillary line (MAL) at the fourth intercostal space; CVP-MTP: CVP measured from the midthoracic plane (MTP) at the anterior fourth intercostal space level.

Of 150 consecutive patients who fulfilled the inclusion criteria and provided informed consent, 93 (62%) subjects were admitted to medical ICU and 57 (38%) to surgical ICU. Patient characteristics are given in Table 1. There was no difference between the mean value, 9.03 (95% CI 8.60–9.46), of CVP-MAL and the mean value, 9.17 (95% CI 8.72–9.62), of CVP-MTP (*p*=0.129). Linear correlation analysis also revealed a strong positive relationship (*r* = 0.908), and the Bland–Altman plot demonstrated a good agreement with the mean difference (mean bias) of near-zero between CVP-MAL and CVP-MTP (Figure 1).

**Figure 2:**
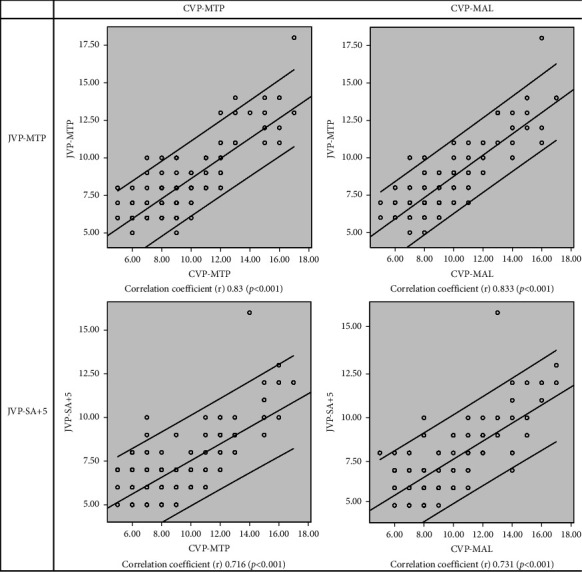
Pearson's correlations of JVP-MTP and JVP-SA + 5 with CVP-MTP and CVP-MAL of 150 subjects. CVP-MAL: central venous pressure (CVP) measured from the midaxillary line (MAL) at the fourth intercostal space; CVP-MTP: CVP measured from the midthoracic plane (MTP) at the anterior fourth intercostal space level; JVP-MTP: JVP measured from the MTP; JVP-SA + 5: jugular venous pressure (JVP) measured from the sternal angle (SA) with the addition of 5 cm.

**Figure 3:**
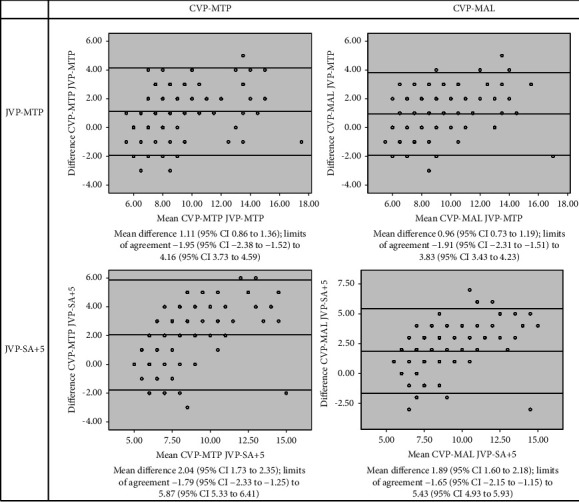
The Bland–Altman plots of JVP-MTP and JVP-SA + 5 with CVP-MTP and CVP-MAL of 150 subjects. CVP-MAL: central venous pressure (CVP) measured from the midaxillary line (MAL) at the fourth intercostal space; CVP-MTP: CVP measured from the midthoracic plane (MTP) at the anterior fourth intercostal space level; JVP-MTP: jugular venous pressure (JVP) measured from the MTP; JVP-SA + 5: JVP measured from the sternal angle (SA) with the addition of 5 cm.

The mean value, 8.07 (95% CI 7.71–8.42), of JVP-MTP was higher than the mean value, 7.13 (95% CI 6.84–7.43), of JVP-SA + 5 (*p* < 0.001). In Pearson correlation analysis, CVP-MTP and CVP-MAL displayed higher correlation coefficients with JVP-MTP than with JVP-SA+5 (Figure 2). The correlation coefficients of CVP-MTP and CVP-MAL with JVP-MTP were almost equal, about 0.83, and with JVP-SA + 5 also almost equal, about 0.72. In Bland–Altman plots also, CVP-MTP and CVP-MAL demonstrated better agreements with JVP-MTP than with JVP-SA + 5 (Figure 3). The mean differences of CVP-MTP and CVP-MAL with JVP-MTP were nearly equal, about 1 cm, and with JVP-SA + 5 also nearly equal, about 2 cm.

## 4. Discussion

In our study, CVP-MTP and CVP-MAL had similar means without significant differences between them and a strong positive linear relationship in Pearson correlation and good agreement with a mean difference of near-zero in the Bland–Altman plot. The similarity between CVP-MTP and CVP-MAL was also independently substantiated by their almost equal degrees of correlation coefficients and mean differences with JVP-MTP and with JVP-SA + 5 also, which were measured separately. The findings indicate that the MAL can be used as the zero-reference level MTP to measure the CVP in the supine position. The practice of measuring CVP in the supine position from the MAL as the external reference point of the zero-reference level is consistent with our finding [1, 9]. The other significant finding in our study is higher correlation coefficients and better agreements of JVP-MTP with CVP-MTP and CVP-MAL than those of JVP-SA + 5. JVP tallied better with CVP when both JVP and CVP were measured from the zero-reference level MTP as the identical external reference point. Variable vertical distances, rather than 5 cm only, between the SA and zero reference level in different reclining positions while measuring the JVP may have caused the lesser degree of correlation and agreement of JVP-SA + 5 with the CVPs measured in the supine position from MTP. The vertical distance between the SA and zero reference level varies in different reclining positions [3].

We also observed in our study that JVP was lower than CVP; that is, CVP was underestimated by JVP, as reported in other studies [1, 2, 13, 16–20]. Measuring CVP with a water column manometer rather than with an electronic transducer could be one reason for its higher values in our study. The CVP values obtained by manometric measurements are, on an average, 2 cm higher as compared to those by the electronic method. This difference is due to a meniscus effect and difficulty identifying mean pressure in the manometer's bobbing saline column [1, 24]. The variation in CVP and JVP is also likely due to the difference in the vertical distances between the external reference points used to measure them in different reclining positions and in the hemodynamic response to the postural change in patients [1, 13].

While interpreting the use of the identical external reference point of the zero-reference level to measure the CVP and JVP in critical care, there are two other factors to consider minimizing the errors in their individual measurements as well as help their matching to the full extent possible. One factor is the ease of accessibility and identification of the external reference point used to measure the JVP and CVP [1, 3, 26]. Though the MTP at the anterior fourth intercostal space level requires measurement in each patient and the MAL at the fourth intercostal space does not require such measurement, both need to be localized in the axilla. However, there is significant variation by several centimeters in the localization of such external reference points of the zero-reference level in the axilla among health care providers, and the variation is not reduced by a laser leveling device [1, 9, 27, 28]. The resultant error in CVP measurement may be equal to or more than the magnitude of a normal CVP value, even in experienced hands [27]. In contrast, the sternal angle (SA) is accessible, identifiable, and easily located reproducibly by physical examination [1–3, 5, 26]. This advantage of SA may have led to its continued use as an external reference point even for CVP measurement [4, 13, 25, 29–32], apart from its use for JVP measurement [1, 5, 13, 26, 33], with the calibration of CVP conducted by further adding 5 cm [4, 30–32] or the vertical distance between SA and MAL [13]. However, whether there is significant variation in the localization of SA or not mostly remains to be studied.

The other readily accessible and identifiable external reference point is the table level. The vertical distance of about 10 cm above the table level in the supine position has also been recommended as the zero-reference level for CVP measurement [6, 34]. This distance is subtracted after measuring the CVP in the supine position from the table level. However, it is not possible to conduct so in higher reclining positions due to the difficulty in defining the point on the table level for CVP and JVP measurement. Otherwise, such distance initially needs to be localized in the supine position in the axilla, where it likes the other external reference points of the zero reference level, may no more be readily accessible and identifiable for routine measurement of CVP and JVP. Apart from the fixed distance, the distance relative to the thoracic diameter from the easily accessible external reference points has also been purposed as the zero-reference level, for example, three-fifths of thoracic diameter from the table level [35] and one-third of thoracic diameter below the SA [6]. However, the relationship of such distances relative to the thoracic diameter with the zero-reference level has not been reported in the higher reclining positions. Moreover, unlike the fixed specified distance, the distance relative to the thoracic diameter from the SA or table level needs to be measured in each patient to add to or subtract from the venous pressure measurement from the SA or table level, respectively. Such measurement requirements in each patient may lead to errors and considerable variability when the method is applied by different or inexperienced personnel [6]. Thus, the distance relative to the thoracic diameter, similar to the fixed distance from the table level, has also been difficult to be used to measure the CVP and JVP, whereas the fixed distance from the readily accessible sternal angle (SA) is continued to be used for JVP [1, 5, 13, 26, 33] and also for CVP [4, 13, 25, 29–32] measurement.

The position of patients is the second factor to consider to minimize the errors in the measurements of CVP and JVP. Though CVP is often measured in the supine position [1, 4, 9, 25, 30–32], it is also measured in higher reclining positions such as 30°, 45°, or even higher up [4, 9, 13, 25, 27]. The midaxillary line (MAL) at the fourth intercostal space is near the zero-reference level in the supine position [1]; however, such a relationship may vary in higher reclining positions [4, 5]. There is hardly any report comparing the CVPs measured in higher reclining positions from the MAL and the zero-reference level MTP. On the other hand, though the vertical distance between the SA and zero reference level is longer in higher reclining patient positions [3], this distance is approximately 5 cm in the supine position [3, 30–32]. Like the CVP measured from MAL, the CVP measured in the supine position from SA with the addition of 5 cm may, thus, match that from MTP. The lesser degree of underestimation of CVP by JVP at lower CVP than the higher degree of underestimation in more elevated CVP [1, 13] indicates such possibility, as the JVP is likely to be measured in the lower reclining positions at lower CVP. Furthermore, the distance between the SA and zero-reference level is more than 5 cm in higher reclining positions; the median vertical distances between the SA and zero reference level are 8, 9.7, and 9.8 cm at 30°, 45°, or 60° elevations, respectively [3]. Though we did not directly compare the CVP and JVP both measured from the SA with the addition of 5 cm, the mean difference between the CVPs in the supine position from MTP and MAL and the JVP-SA + 5 was also about 2 cm in our study. Had a higher number been added to the JVP measurement, instead of 5 cm only, from the SA, it could have also matched better with the CVPs. In higher reclining patient positions, good correlation and agreement may also be found between the CVP-MTP and the CVP measured from the sternal angle (SA) along with the addition of higher numbers, instead of 5 cm only, for different such positions. There is, however, hardly any report comparing the CVP measured from MTP with the CVP from SA along with the addition of 5 cm for supine or of higher numbers, for example, 6, 7, or 8 cm, for higher reclining, e.g., 30°, 45°, or 60°, patient positions.

Further study of CVP and JVP measurements in similar reclining positions from various identical external reference points is warranted to explore the matching of CVP and JVP to the full extent possible. Our findings indicate that such a study is possible by standardizing the measurements of CVP and JVP examined from other identical external reference points to that from the zero-reference level MTP. Future researchers should, thus, consider a larger study of CVP and JVP with their measurements by various valid methods from different identical external reference points, for example, MTP, MAL, and SA, as previously discussed, and in corresponding reclining positions, such as 0°, 30°, 45°, and 60°, with the objectives of clarifying the correlations and agreements between them as well as their individual variations with different external reference points.

Our study had a unique design with CVP measurement from two external reference points and JVP from two external reference points to compare different venous pressure measurements in a single study. To analyze the agreement between the venous pressures, we also used the Bland–Altman plot [36], which was not used in many studies comparing CVP and JVP [14–20]. Our study also has several limitations. We previously discussed the measurement of CVP by a water column manometer as a limitation of the study. Next, the ICU physicians measured the CVP from MTP and MAL, and the first author measured the JVP from MTP and SA. As the CVP and JVP were measured in a blinded fashion, almost equal degrees of correlation coefficients and mean differences of CVP-MTP and CVP-MAL with JVP-MTP, and with JVP-SA + 5 as well, substantiate the CVP and JVP measurement findings independently recorded by the two groups. Then, almost three-fourths of the patients in our study had the right subclavian catheters. An intravenous line in the neck often obscures the observation of jugular venous pulsations [5]. Thus, the inclusion of the patients with visible right internal jugular venous pulsations in our study may have caused the selection of a lesser proportion of patients with an internal jugular catheter and a higher proportion with a subclavian catheter. It may be difficult to identify the jugular veins in 6% to 28% of patients, especially those with an internal jugular catheter that may obscure the neck veins [1]. However, all the participants in our study had both CVP and JVP data due to the inclusion of patients with visible right internal jugular vein pulsations. The number of participants in our study was also more than the numbers, 25 to 103, in previous studies comparing CVP and JVP [13–20]. Whether there is any difference between the CVP measured through a subclavian catheter and that through an internal jugular catheter has not been reported since two such central venous catheters are seldom inserted simultaneously in one subject. The strong correlation and good agreement of the CVP measured through a peripherally inserted central venous catheter with that through a centrally inserted central venous catheter measured simultaneously in the same subjects indicates the possibility that there may not be such a difference [37].

## 5. Conclusions

JVP tallies better with the CVP examined in the supine position when both are measured from the zero-reference level MTP as the identical external reference point, and MAL can be used to measure the CVP in the supine position. Our findings indicate the way to explore the matching of CVP and JVP to the full extent possible by standardizing their measurements from other external reference points to that from the zero-reference level MTP. We discussed that even with the use of the identical external reference point (ERP) to measure the CVP and JVP, the two other factors that can affect their measurements and matching are the accessibility of the ERP and the reclining angles of the patients. We also previously discussed the difficulties in localizing the ERPs in the axilla and in using the table level in higher reclining positions and the distances relative to the thoracic diameter as ERPs for measuring the venous pressures in the day-to-day clinical practice. The obvious accessibility of the sternal angle (SA) may also be studied. However, there is hardly any report of comparing the CVP measured in higher reclining positions from the zero-reference level MTP with that from the MAL and from the sternal angle (SA) with the addition of 5 cm or higher numbers. The vertical distance between the SA and zero-reference level (ZRL) is about 5 cm in the supine position but longer in higher reclining positions where the nearer relationship of MAL with the ZRL may also vary. As indicated by our findings, further study of CVP and JVP measurements in similar reclining positions from identical ERPs such as MTP and MAL and the sternal angle with the addition of higher numbers for higher reclining positions instead of 5 cm only is warranted standardizing other measurements to that from MTP. Such standardization with the zero-reference level MTP will help clarify the correlations and agreements between the CVP and JVP measurements as well as their individual variations in similar reclining positions in relation to different external reference points.

## Figures and Tables

**Table 1:** Characteristics of 150 subjects.

Characteristics	Value
Age and mean ± SD (years)	50.67 ± 18.96
Male/female ratio	79/71
Nonsmoker/smoker ratio	95/55
Body mass index and mean ± SD (kg/m^2^)	23.64 ± 3.41
ICU diagnosis, no.	
Medical ICU	
(1) Sepsis	20
(2) Pneumonia	16
(3) Congestive cardiac failure	15
(4) Poisoning	12
(5) Decompensated chronic liver disease	5
(6) Pancreatitis	4
(7) Acute respiratory distress syndrome	3
(8) Miscellaneous	18
Surgical ICU	
(1) Postoperative case	46
(2) Pancreatitis	1
(3) Acute respiratory distress syndrome	1
(4) Miscellaneous	9
Right subclavian catheterization, no.	109
Right internal jugular catheterization, no.	41
CVP-MTP, mean ± SD (cm H_2_O)	9.17 ± 2.79
CVP-MAL, mean ± SD (cm H_2_O)	9.03 ± 2.65
JVP-MTP, mean ± SD (cm H_2_O)	8.07 ± 2.25
JVP-SA + 5, mean ± SD (cm H_2_O)	7.13 ± 1.87

CVP-MAL: central venous pressure (CVP) measured from the midaxillary line (MAL) at the fourth intercostal space; CVP-MTP: CVP measured from the midthoracic plane (MTP) at the anterior fourth intercostal space level; JVP-MTP: jugular venous pressure (JVP) measured from the MTP; JVP-SA  +  5: JVP measured from the sternal angle (SA) with the addition of 5 cm.

## Data Availability

The datasets used for analysis in the current study are available from the corresponding author upon reasonable request. The analyzed results of the datasets of this study are included within the article.

## Abbreviations

CVP: Central venous pressure
CVP-MAL: CVP measured from MAL
CVP-MTP: CVP measured from MTP
ERP: External reference point
JVP: Jugular venous pressure
JVP-MTP: JVP measured from MTP
JVP-SA + 5: JVP measured from SA with the addition of 5 cm
MAL: Midaxillary line at the fourth intercostal space
MTP: Midthoracic plane at the anterior fourth intercostal space level
SA: Sternal angle
ZRL: Zero-reference level.
